# Indirect organogenesis from leaf explants of *Eucalyptus benthamii x Eucalyptus dunnii* and shoot multiplication

**DOI:** 10.1186/1753-6561-5-S7-P145

**Published:** 2011-09-13

**Authors:** Yohana de Oliveira, Laís Gomes Adamuchio, Cassiana de Oliveira, Juliana Degenhardt-Goldbach, Isabel Gerhardt, João Carlos Bespalhok Filho, Roberson Dibax, Marguerite Quoirin

**Affiliations:** 1 Departamento de Fitotecnia e Fitossanitarismo, Pós-Graduação em Agronomia-Produção Vegetal, Universidade Federal do Paraná (UFPR), 80035-050, Curitiba, Paraná, Brazil; 2 Graduação em Agronomia, UFPR, Curitiba, Paraná, Brazil; 3 Departamento de Fitotecnia e Fitossanitarismo, Pós-graduação em Agronomia-Produção Vegetal, UFPR, Curitiba, Paraná, Brazil; 4 Embrapa Florestas, Estrada da Ribeira, km 111, Caixa Postal 319, Colombo, Paraná, Brazil; 5 Embrapa Florestas, Colombo, Paraná, Brazil; 6 Departamento de Fitotecnia e Fitossanitarismo, Pós-graduação em Agronomia-Produção Vegetal, Universidade Federal do Paraná (UFPR), 80035-050, Curitiba, Paraná, Brazil; 7 Departamento de Botânica, UFPR, Caixa Postal 19031, 81531-990, Curitiba, Paraná, Brazil

## Background

In Brazil, especially in the Southern region, stresses caused by cold and eventual frost are those that exert the most negative effect on the productivity of *Eucalyptus* spp. The genetic transformation techniques may contribute to forestry improvement programs in order to obtain genotypes expressing new interesting characteristics. They allow shortening the long breeding cycles and avoiding manipulation of adult trees. Their efficiency depends on establishment of regeneration procedures that allow the development of shoots from the transformed tissues.

*E. benthamii*x *E. dunnii* hybrids have shown superiority to their parents concerning growth and frost tolerance [1], but no information about their *in vitro* organogenesis has been reported in the literature.

The objective of this study was to evaluate the effect of some factors of culture medium on indirect organogenesis and shoot multiplication of an*E. benthammi* x *E. dunnii* clone.

## Methods

*In vitro*established shoots,provided by EMBRAPA-Florestas (Colombo, PR, Brazil), were used as explant source. Cultures were maintained under white fluorescent tubes providing a photon flux density (PFD) of approximately 20 μmol m^-2^ s^-1^, a 16-h photoperiod and a temperature of 25±2 °C.

The cultures were performed in glass flasks containing 25 mL MS [2] medium supplemented with 1.11µM BA and sealed with rigid polypropylene caps. For the indirect organogenesis, leaves were excised from shoots at the petiole base, split into two halves and inoculated in culture media. The cultures were done in Petri dishes kept in a growth chamber in the dark throughout the experiment.

The statistical design was performed in a factorial scheme (2:2:2) and a comparison was done between two culture media (MS-N/2, with half concentration of potassium and ammonium nitrates, and JADS [3] with 0.1 µM NAA, with and without PVP-40 (250 mg L^-1^) and two TDZ concentrations (0.1 and 0.5 µM).

After 70 days, the percentages of explants forming callus, oxidized explants, explants producing anthocyanin, explants forming buds and shoots, and the number of shoots per explant were evaluated.

For the multiplication test, the statistical design was performed in a factorial scheme (3:2) with three culture media (MS, WPM [4] and JADS, with 1.11µM BA) and two subcultures (28 and 56 days after the initial culture period). The analyzed variables for each subculture were: percentage of oxidation, of explants showing chlorosis, fresh weight and number of shoots.

## Results and discussion

Regarding the oxidation, the higher rates (100%) were observed on JADS medium in presence or absence of PVP-40 and on MS-N/2 medium (68.3%) in presence of PVP-40. However, the JADS medium showed the highest percentage of callus formation (83.3%). In MS-N/2 medium the highest percentage of callus formation (55%) was found in the presence of PVP-40 and 0.5µM TDZ. Similar results were observed [5] with *E. saligna* leaf explants cultured on MS-N/2 medium supplemented with 0.1µM NAA and 1.0µM TDZ. Anthocyanin was only observed in explants cultured on MS-N/2 medium without PVP and containing 0.5µM TDZ. The lowest percentage of explants presenting anthocyanin was observed on MS-N/2 with PVP-40 that gave the best result for organogenesis (8.3%).

For the multiplication experiment, the variance analysis did not reveal significant interaction between the factors. During the first subculture MS medium with 1.11µM BA promoted the highest number of shoots/explant (9.2).The explants cultured on MS medium showed the highest fresh weight (0.384g) and highest percentage of chlorotic explants (88%). The percentage of oxidized explants was the same on JADS and MS media (40%). In a study on *E. urophylla* x *E.* g*lobulus* shoot multiplication [6], an increase of oxidation was also observed on JADS medium in comparison with MS medium.

With regard to the second subculture, the number of shoots per explant and percentage of oxidation did not differ among the treatments. The explants cultured on MS medium showed the same behavior for the chlorosis than during the first subculture and the percentage of chlorotic explants was superior to that observed in others treatments (64%). On WPM medium, the mean number of shoots/explant was the highest (9.08).

## Conclusions

*E. benthamii*x *E. dunnii* hybrid has a low organogenic response to the tested combinations of growth regulators and high tendency to oxidation even when an antioxidant is present in the culture medium. However, the concentration of PVP-40 used in this study may have been low and other types of antioxidants and concentrations need to be compared.

The MS medium with 1.11 µM BA promoted the highest number of shoots per explant after a 28 day culture period, and after 56 days, there were no differences among the treatments.

## Acknoledgements

The authors thank CNPq (Conselho Nacional de Pesquisa, Brazil) and Fundação Araucaria (Parana State, Brazil) for funding the research.
